# Precision Mental Healthcare: Identifying Service Preferences Through Discrete-Choice Experiments in Chinese Megacities

**DOI:** 10.1007/s10488-025-01444-z

**Published:** 2025-04-22

**Authors:** Juan Chen, Luqi Yuan, Bo Li, Jie Yan, Liying Ren

**Affiliations:** 1 Department of Applied Social Sciences, The Hong Kong Polytechnic University, Hung Hom, Kowloon, Hong Kong China; 2 Mental Health Research Centre, The Hong Kong Polytechnic University, Hung Hom, Kowloon, Hong Kong China; 3 School of Government, Peking University, No. 5 Yiheyuan Road, Haidian District, Beijing, 100871 China; 4 Institute of Sociology, Chinese Academy of Social Sciences, No. 5 Jianguomennei Street, Dongcheng District, Beijing, 100732 China

**Keywords:** China, Decision-making, Help seeking, Mental health, Service use, Survey experiments

## Abstract

Designing mental health services that align with individual preferences is a cornerstone of patient-centered care, enhancing both service utilization and treatment outcomes. This study applied the precision framework of mental healthcare to explore preferences for first-contact mental health services among community residents and family members with mild psychiatric symptoms in Chinese megacities. Using a discrete choice experiment, an online survey was conducted with 4,057 participants from Beijing, Shanghai, Guangzhou, and Shenzhen. Mixed logit analysis identified service providers as a key determinant of mental health service utilization across cities. While both potential patients and family members favored lower-cost public services, notable variations emerged in preferences for psychological counseling/therapy across cities and between patient and family contexts. Latent class analysis further revealed four distinct preference groups among potential patients. Psychological distress levels and hukou type were significantly associated with preferences. These findings highlight the importance of tailored interventions that accommodate patient and family needs, leveraging the unique features of China’s mental healthcare system to improve access, quality, and equity in mental health services.

**Supplementary Information:**

The online version contains supplementary material available at 10.1007/s10488-025-01444-z.

## Introduction

Mental health in Mainland China is an escalating concern, driven by economic and societal pressures. The COVID-19 pandemic further intensified these challenges, with psychological distress surging to affect nearly 35% of the population (Qiu et al., 2020). This highlights critical weaknesses in China’s mental healthcare system, underscoring the need for reforms to enhance resilience and ensure long-term mental well-being.

Mental health services in China are marked by a pronounced urban-rural divide, with significant disparities in resource allocation, accessibility, and service quality (Zhang et al., 2022). Urban centers like Beijing and Shanghai benefit from advanced mental healthcare infrastructures that include specialized hospitals, well-established community health centers, and growing access to professional counseling (Chen, 2012). In contrast, rural areas struggle with severe shortages of trained professionals and limited services, often relying on general practitioners with minimal psychiatric training (Zhang et al., 2022).

To address these inequalities, the Chinese government has implemented policies aimed at improving nationwide access to mental healthcare. The National Mental Health Work Plan (2015–20) prioritized integrating mental health into primary care, while the Healthy China 2030 strategy seeks to bridge the urban-rural divide through equitable healthcare access (Zhong & Wang, 2021). Additionally, the 686 Program, launched in 2004, has played a pivotal role in providing free treatment for severe mental illnesses and expanding community-based services (Liang et al., 2018). These initiatives reflect a significant commitment to reducing disparities and strengthening the mental healthcare system.

While these policies have improved service availability, significant gaps persist in meeting the diverse mental health needs of megacity residents (Chen, 2018). Disparities in service quality and accessibility across districts within the same city hinder equitable care (Liang et al., 2018). Additionally, the pervasive stigma surrounding mental illness continues to deter help-seeking, especially among high-risk groups such as migrants and low-income individuals (Hu et al., 2017; Yu et al., 2015). These challenges underscore the need for region-specific insights to inform more targeted and effective mental health policies and interventions.

In urban China, community health facilities serve as key providers of mental health services (Li et al., 2023), primarily focusing on monitoring and rehabilitation for individuals discharged from psychiatric hospitals or treatment centers. However, outreach programs, referral services, and social support for families and children remain lacking (Chen, 2018).

Despite efforts to develop community-based, patient-centered psychiatric care, professional help-seeking for mental health issues remains rare in China. Many delay treatment for years after symptom onset, while others never seek care (Chen, 2012, 2018). This underscores the urgent need to expand professional mental health services and increase the availability of well-trained practitioners (Hu et al., 2017). However, without addressing barriers to care, improvements in service availability and quality will yield limited benefits. Understanding preferences and decision-making processes in mental health service use is crucial to reducing these barriers and improving access to effective care (Chen, 2018).

Recent studies reveal that preferences for mental health services in China are shaped by a complex interplay of cultural, structural, and economic factors (Chen, 2018; Qin & Hsieh, 2023; Yu et al., 2015; Zhong & Wang, 2021). Public mental health services are often preferred for their affordability and perceived reliability, particularly in urban areas offering greater availability (Zhong & Wang, 2021). Emerging digital interventions, such as online counseling platforms and AI-powered assessment tools, are increasingly popular, especially among younger, tech-savvy individuals, due to their privacy, flexibility, and cost-effectiveness in a context where traditional counseling may be stigmatized or logistically challenging (Bazarkina et al., 2023). Additionally, holistic approaches incorporating Traditional Chinese Medicine are gaining traction, reflecting cultural values that emphasize natural and integrative treatments (Lu et al., 2022). These shifting preferences highlight the need for mental health policies and interventions tailored to the diverse needs and contexts of China’s population.

This study explores mental health service preferences in four major Chinese megacities—Beijing, Shanghai, Guangzhou, and Shenzhen—within the framework of precision mental health. Using a discrete choice experiment methodology, it evaluates the relative importance of service attributes, including cost, provider type, family involvement, and delivery format. By examining individual and family decision-making processes, the study addresses critical gaps and provides insights into barriers and facilitators of mental healthcare use.

This study employs discrete choice experiments to examine mental health service preferences in Chinese megacities. Unlike prior research that relies on qualitative interviews or quantitative surveys (e.g., Chen, 2018; Han et al., 2018; Tan et al., 2021; Yu et al., 2015), it systematically assesses the relative importance of service attributes. By analyzing preferences across four major urban centers, this study provides empirical insights into localized mental health needs and establishes a transferable methodological framework for designing patient-centered interventions. These findings address gaps in understanding patient and family preferences, offering evidence-based strategies to enhance the accessibility and effectiveness of mental health services in China’s evolving healthcare landscape.

### Precision Mental Health

The concept of precision first gained prominence in clinical settings, where it became central to tailoring therapeutic interventions to individual patient characteristics (König et al., 2017). While precision medicine excels at addressing specific biological risk profiles, its narrow focus on individual biomedical factors limits its applicability to broader public health challenges. This approach often neglects the social and psychological determinants of health, prompting growing recognition of the need for a more holistic framework that integrates sociomedical factors to address the full spectrum of health determinants.

Precision public health has emerged to bridge this gap, shifting the focus from individual patients to entire populations as the target of intervention (Olstad & McIntyre, 2019). It acknowledges that health challenges extend beyond the biomedical domain, shaped by sociocultural, economic, and environmental factors. By integrating biosocial strategies, precision public health aims to address these complex determinants and improve population-wide well-being.

Precision public health is an innovative approach to improving population health, defined by two core attributes. First, it leverages technological advancements (Horton, 2018). For example, the UK’s national green policy highlights the use of smartphone applications for targeted behavioral interventions (UK Government, 2019), while the global COVID-19 response demonstrated its effectiveness in integrating technology into public health strategies (Moore et al., 2023). Second, precision public health is data-driven (Canfell et al., 2022), utilizing extensive datasets and continuous information streams, including genomic data, spatial profiling, and social determinants of disease risk (Khoury, 2018). By combining advanced technology with robust analytics, precision public health delivers tailored interventions that address diverse population needs in specific contexts (Khoury, 2018).

Chinese cities have embraced precision public health to improve population health outcomes. In Shanghai, the “Smart Health Posthouses” system integrates data from wearable devices, electronic health records, and community health centers (Zhang et al., 2021). This platform monitors chronic conditions such as hypertension and diabetes, enabling tailored health interventions to address individual needs.

Mental healthcare has adopted precision methodologies to enhance its effectiveness. Precision mental health shifts the focus to data-driven prevention and intervention strategies, emphasizing comprehensive assessments of individual needs, preferences, and prognostic factors (Bickman et al., 2016). Through detailed evaluations, continuous monitoring, and customized feedback, it provides personalized support (Chen et al., 2024). Building on precision public health principles, precision mental health integrates technology, data analytics, and tailored interventions to address mental health challenges (Scala et al., 2023).

China has recently developed precision mental health services targeting specific demographics. In Shenzhen, an AI-powered assessment system identifies students at risk of anxiety or depression by analyzing survey data, behavior patterns, and academic performance, providing tailored recommendations for psychological support and counseling (Bazarkina et al., 2023).

Precision mental health has garnered increasing academic attention for its transformative potential (Chen et al., 2024; Purgato et al., 2021; Szatmari & Susser, 2022). However, much of the discourse remains theoretical, with limited empirical research, particularly regarding service preferences—a key component of precision mental health (Bickman et al., 2016). This study fills that gap by examining mental health service preferences among adults in four Chinese megacities: Beijing, Shanghai, Guangzhou, and Shenzhen.

These cities are notable for their advanced community mental healthcare systems, reflected in key metrics. Beijing and Shanghai lead with 2123 and 1,191 community health organizations, supported by workforces of 35885 and 38256 community health workers—far exceeding national averages of 53 organizations and 996 workers per city (Beijing Municipal Bureau of Statistics, 2023; National Health Commission, 2023; Shanghai Municipal Bureau of Statistics, 2023). Although Guangzhou and Shenzhen have fewer organizations (333 and 841, respectively), they maintain substantial infrastructures with 16800 and 15160 community health workers (Guangzhou Municipal Health Commission, 2024; Public Hygiene and Health Commission of Shenzhen Municipality, 2024). These resources underscore the high quality and accessibility of community mental health services in these cities, distinguishing them from others in China.

Renowned for their advanced healthcare provision, these urban centers provide an ideal context for studying service preferences. While the findings are context-specific, the robust infrastructure in these cities offers valuable insights for scaling and adapting similar initiatives across other regions in Mainland China. The study’s outcomes aim to guide efforts to optimize mental health service delivery nationwide, ensuring a more effective and context-sensitive implementation of precision mental health principles.

### Research Questions

Developing mental health interventions that align with patient preferences is vital for patient-centered care, as it boosts both service uptake and treatment outcomes (Becker et al., 2016). Sustainable mental health services must also incorporate the preferences of patients and families (Becker et al., 2016). However, most studies on preferences in Chinese mental healthcare have relied on qualitative interviews (e.g., Chen, 2018; Tan et al., 2021; Yu et al., 2018) or quantitative measures of attitudes and beliefs (e.g., Chen, 2012; Han et al., 2018; Yu et al., 2015). These methods fall short in reliably and comprehensively evaluating the relative importance of key factors (Shi et al., 2020).

This study employed a discrete choice experiment design to conduct an online survey among community residents in Beijing, Shanghai, Guangzhou, and Shenzhen. The research addresses two key questions: (i) What factors influence community residents and their families in choosing first-contact mental health services for mild psychiatric symptoms? (ii) What strategies can overcome barriers to accessing and utilizing mental health services, ensuring equitable and effective care? By tackling these questions, the study offers actionable insights into mental health service utilization, highlights opportunities to optimize resource allocation, and strengthens mental healthcare systems in Mainland China. These questions provide a focused framework for understanding the study’s findings and implications.

## Methods

### Design

A discrete choice experiment is a factorial survey method used to elicit preferences for service, intervention, or product features (Becker et al., 2016). By presenting respondents with hypothetical scenarios containing choice profiles with varying attribute levels, it quantifies the relative importance of factors influencing decision-making (Chen et al., 2024; Lancsar & Louviere, 2008).

### Attributes and Scenarios

#### Attribute Selection

We began by conducting a thorough review of policy documents and news articles to assess the current state of mental health services in Mainland China, which informed our selection of study attributes. An extensive literature review further identified factors influencing mental healthcare utilization and help-seeking behaviors, leading to the development of a preliminary list of potential attributes and levels. This list was refined through feedback from mental health experts, resulting in the rewording, merging, or removal of certain attributes to ensure contextual relevance. Ultimately, seven attributes were selected (Table 1).

**Table 1:** Attributes and levels

Attributes	Levels	Explanation
Service providers	1) Community health service center/station	Organizations/facilities offering mental health services
	2) Social service organization	
	3) Public hospital	
	4) Private clinic/hospital	
Recommendation sources	1) Public education/promotion program	Sources from which respondents obtain reliable information about available mental health services, indicating their familiarity with these services
	2) Friends/family	
	3) Past users	
	4) Health professionals	
Service formats	1) Online/mobile app	Service delivery methods
	2) Telephone/hotline	
	3) Face-to-face/in-person	
Individual/group consultations/treatments	1) Individual consultation/treatment	Whether consultation or treatment is provided individually or in a group setting
	2) Group consultation/treatment	
	3) Choice of individual/group consultation/treatment	
Intervention types	1) Alternative treatments (e.g., exercise, acupuncture, massage therapy)	Treatment modalities and options used by care providers
	2) Lecture/course	
	3) Psychological counseling/therapy	
	4) Pharmacological treatment/medication	
Out-of-pocket costs per visit (RMB)	1) Free	Out-of-pocket medical expenses, including deductibles, coinsurance, and copayments, not covered by insurance or government subsidies
	2) 50	
	3) 250	
	4) 500	
Family involvement	1) Not always involved	Whether family involvement is required in the consultation or treatment process
	2) Always involved	

Research highlights that structural barriers—such as high costs, inadequate information about available services, and limited awareness of treatment options—pose significant obstacles to help-seeking and are key predictors of mental health service utilization (Chen, 2012, 2018; Han et al., 2018). Addressing these barriers can support healthcare providers and policymakers in designing services that are more accessible, affordable, and effective. Table 2 outlines the rationale and empirical evidence supporting the selection of these attributes.

**Table 2:** Rationale for attribute selection

Service providers	Research highlights significant variation in Chinese residents’ preferences for healthcare facilities (Chen, 2018; Qin & Hsieh, 2023; Yu et al., 2015). Studies show a general preference for large, publicly funded hospitals over primary care or private facilities for mental health treatment (Zhong & Wang, 2021). For instance, a survey in Tianjin found that psychiatric hospital clinics were the most used mental healthcare resource, while community health service centers were rarely utilized by individuals with psychological distress (Yin et al., 2019). Similarly, Beijing respondents reported infrequent use of community health service centers for psychological support (Chen, 2012). Low utilization rates of community health services may stem from residents’ doubts about service quality (Li et al., 2023). Additionally, a discrete choice experiment revealed that preferences for healthcare facilities in treating general illnesses varied by hukou status (Tang et al., 2016). These findings underscore the need to investigate the types of providers preferred by individuals with psychological symptoms. Understanding these preferences can guide policy decisions and optimize resource allocation.
Recommendation sources	Structural barriers, particularly low accessibility, remain a significant deterrent to mental health service utilization. Many individuals, despite their willingness to seek help from mental health professionals, report uncertainty about where to access services or assume such services are unavailable (Chen, 2018; Han et al., 2018; Yu et al., 2015). This lack of information about accessible services often delays treatment. Notably, older adults and individuals experiencing severe psychological distress perceive accessibility barriers more acutely than others (Chen, 2012). Potential users may obtain reliable information about mental health services through passive methods, such as public awareness campaigns, or active approaches, including consultations with health professionals, discussions with family or friends, or advice from peers with similar experiences (Gearing et al., 2024).
Service formats	Research indicates that telephone and Internet-based mental health interventions can alleviate depression symptoms (Kim et al., 2020). Online services and hotlines have gained popularity as resources for mood disorders, offering significant advantages such as flexibility, lack of physical constraints, and a high degree of confidentiality. However, China’s digital divide may hinder widespread adoption of Internet-based mental health treatments (Chen & Zhu, 2016). Online interventions are particularly suitable for specific populations, including educated young adults, individuals with higher socioeconomic status, urban-to-urban migrants, and psychologically distressed subgroups. These groups have shown a stronger preference for utilizing online resources compared to others (Chen & Zhu, 2016). Within psychologically distressed populations, individuals with mild to moderate anxiety or depression are more likely to benefit from tailored Internet-delivered treatments than those experiencing severe symptoms (Gun et al., 2011).
Individual/group consultations/treatments	Group psychotherapy offers a cost-effective alternative to one-on-one psychotherapy for conditions like primary insomnia. However, some clients hesitate to participate in group programs, especially in community-based settings, due to concerns about neighbors discovering their mental issues (Yu et al., 2018). Additionally, increasing the number of participants in a group can diminish the quality of interactions. In contrast, individual treatment provides personalized attention in a private setting, alleviating concerns about confidentiality or peer judgment. Research suggests that group therapy is best suited as an initial intervention within a stepped-care approach to mental health services, rather than as a replacement for one-on-one treatment (McDermut et al., 2001).
Intervention types	Growing evidence suggests that prolonged delays in seeking mental healthcare often stem from inadequate information about treatment options and fear of medication (Han et al., 2018). In Singapore, mental health professionals view them as beneficial, whereas approximately 40% of potential users perceive them as harmful (Picco et al., 2016). Research also indicates a strong preference among patients for psychological therapy over pharmacological interventions (Andrade et al., 2014). Similarly, alternative treatments like exercise enjoy widespread support among potential users. However, it remains questionable whether these approaches alone can effectively address persistent depression or anxiety without a structured treatment plan.
Out-of-pocket costs	Structural barriers, such as financial constraints, are significant deterrents to seeking mental health services. Studies have shown a strong link between medical costs and the intention to seek help, particularly among individuals with greater psychological distress or a recognized need for treatment (Han et al., 2018). In Beijing, some respondents viewed professional mental health services as a form of conspicuous consumption, further discouraging utilization (Chen, 2018). Additionally, economically disadvantaged individuals are found to be more sensitive to service costs compared to those with higher socioeconomic status (Chen, 2012).
Family involvement	Research suggests that families are often a preferred and accessible source of support for individuals experiencing mental disorders, particularly young people (Chen, 2012; Yin et al., 2019). However, families can either facilitate or hinder help-seeking behavior and the effectiveness of professional treatment (Chen, 2018). A lack of understanding about mental illness and limited family involvement in treatment may contribute to families undervaluing the role of mental health professionals.

#### Hypothetical Choice Scenarios

Each discrete choice experiment task presented a hypothetical scenario tailored to respondents’ roles—either potential patients or family members—to capture diverse service preferences. Potential patients were asked, “Which service would you prefer for first-contact care when experiencing mild psychiatric symptoms?” Family members, meanwhile, selected the service they deemed most appropriate for a relative facing mild mental health issues.

For each scenario, respondents selected between Service A, Service B, or an opt-out option, indicating a preference to forgo professional services and maintain the status quo. The opt-out option was included to mitigate the risk of overestimating attribute influence (Lancsar & Louviere, 2008).

### Experimental Design

A full factorial design would have generated 4,608 choice profiles across seven attributes—four with four levels, two with three levels, and one with two levels—making survey implementation impractical. To address this, we used a fractional factorial design to select a manageable subset while maintaining feasibility and flexibility (Chen et al., 2024). Using SAS, we generated a reduced yet effective design, identifying 96 balanced and orthogonal combinations (Kuhfeld, 2005).

We conducted a pilot test with 100 participants to refine the final experiment design. A fractional factorial design was generated in Ngene using zero priors, assuming all parameter priors were equal to zero (Bliemer et al., 2008).

During the pilot test, we assessed task clarity, comprehension, and ease of understanding. Ambiguous or overly complex wording was identified and revised to ensure respondents accurately interpreted the choice tasks and attributes. We also analyzed response patterns, such as repetitive selections, to detect potential misunderstandings and confirmed that attribute level coefficients aligned with expectations. Based on these findings, we refined the survey by adding a nontechnical introduction and simplifying choice questions to enhance clarity and validity.

Before launching the main survey, we optimized the experimental design using pilot data estimates as prior inputs. A D-efficient Bayesian design was then generated using a multinomial logit model with 1000 Halton random draws.

**Fig. 1:**
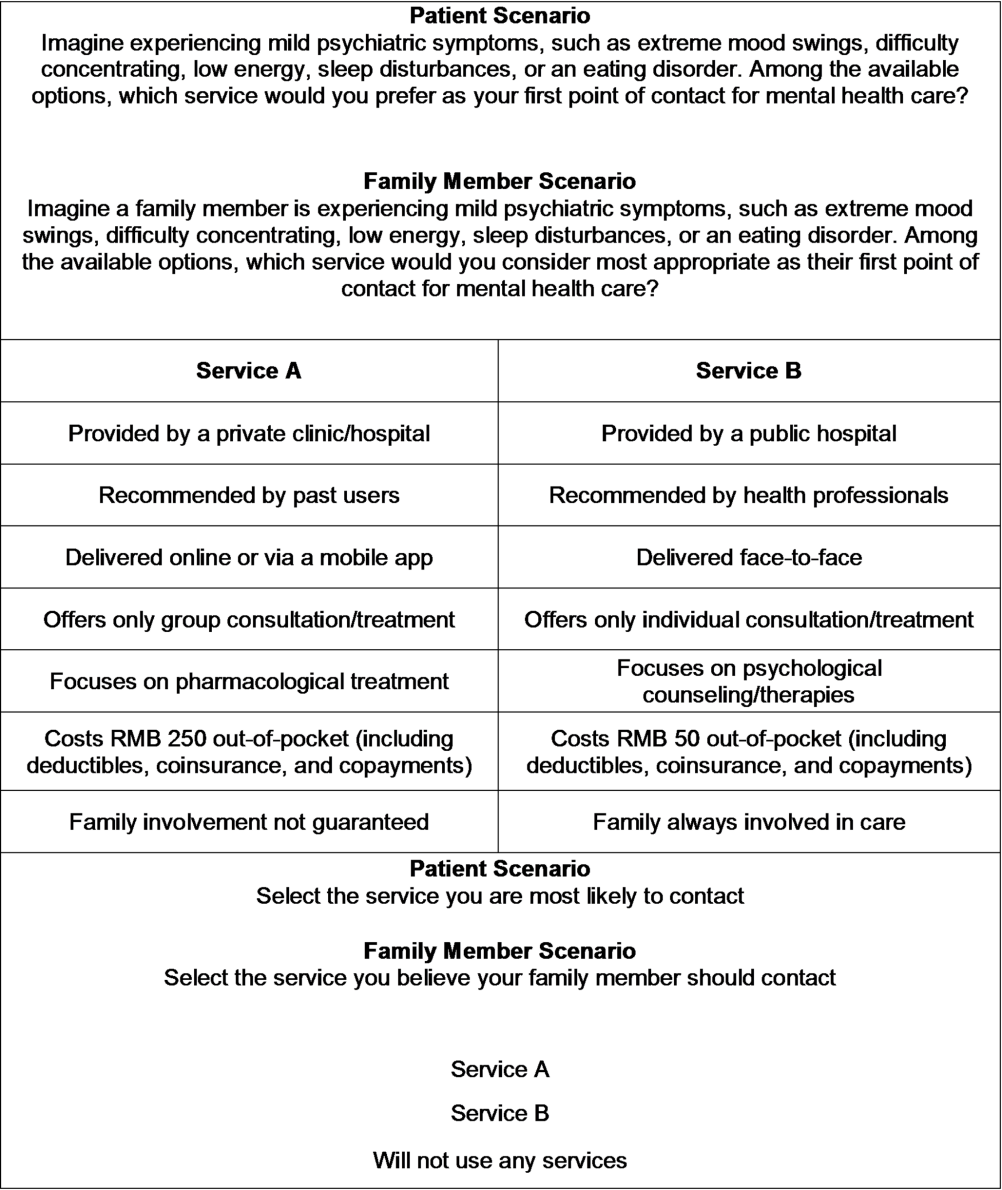
Sample discrete choice experiment choice set

The survey included 48 pairs of choice tasks (96 choice profiles) for scenarios involving potential patients and family members, divided into four subsets of 12 pairs each using Ngene’s block design (Bridges et al., 2011). The final questionnaire was selected based on the design with the smallest D-error after convergence, with no further improvements observed within 15 min. Respondents were randomly assigned one of the four subsets. Figure 1 illustrates an example of a discrete choice experiment task.

### Data Collection

The survey, conducted from December 2022 to March 2023, targeted Chinese adults aged 18–75 who had lived in Beijing, Shanghai, Guangzhou, or Shenzhen for at least 6 months. Participants were recruited via the survey agency Yipai using an online panel, with a target of at least 1,000 respondents per city to ensure robust estimates. Quota sampling was applied to achieve representativeness across gender (50% female, 50% male) and age groups (18–24: 20%; 25–34: 20%; 35–44: 20%; 45–54: 20%; 55–75: 20%).

Founded in 2004, Yipai is a leading provider of online survey development and implementation in China. Its advanced ePanel platform (https://www.epanel.cn), supported by an experienced technical team and a high-quality online sample library, has facilitated nationwide studies for top-tier universities such as Peking and Tsinghua. With nearly two million individuals in its database, Yipai offers extensive coverage, particularly in major cities, ensuring broad representativeness for our survey.

Yipai’s database includes a wide range of demographic variables, such as location, gender, age, marital status, education, occupation, income, and housing details. This diversity allows for precise quota sampling on key variables like age and gender, ensuring respondent distributions align with local population demographics.

Yipai employs a rigorous quality control system that oversees every stage of the survey process—from questionnaire design and respondent recruitment to data retrieval and cleaning. This system minimizes human error, enabling efficient large-scale data collection while ensuring data reliability.

A total of 4057 respondents completed the survey, with quality control measures ensuring data integrity. During the cleaning process, we identified inconsistencies, such as mismatched age and education details. For the analysis, we ensured proper coding of attribute levels and accurate data transformations.

We first reshaped the responses from wide to long format, with each choice set represented by three rows: Service A, Service B, or the opt-out option. The data were then converted to binary format, coding the choice variable as 1 for selected alternatives and 0 for non-selected ones. These responses were combined with the attribute levels from the Ngene-generated choice profiles in preparation for analysis.

We also examined extreme choice patterns, such as repetitive selections based on a single attribute, to ensure respondents were meaningfully engaging in trade-offs. Forty-two respondents were excluded due to inconsistencies in residency information, leaving 4015 valid questionnaires for analysis. Poststratification weights were applied to adjust the sample to align with local population distributions for key variables like age and gender.

Mental health status in the survey was assessed using the Kessler Psychological Distress Scale (K6), a 6-item tool measuring the frequency of non-specific mental distress over the past month (Kessler et al., 2002). The K6 scores were summed to produce a total score ranging from 0 to 12. Tables 3 and 4 present descriptive statistics on the demographic characteristics, socioeconomic status, mental health, and medical insurance coverage of the study sample, alongside gender and age data from the 2020 China National Population Census for the four cities.

**Table 3:** Descriptive statistics of the study sample (Beijing and Shanghai)

Variables	Beijing ^a^			Shanghai ^b^		
	Mean/percentage	Poststratification weighted mean/percentage	2020 China Census	Mean/percentage	Poststratification weighted mean/percentage	2020 China Census
*Demographic characteristics*						
Age (18–75, mean)	41.13 (0.48)	43.09 (0.49)		41.22 (0.49)	45.03 (0.53)	
Age groups (%)						
18–24	20.89%	9.20%	9.41%	20.94%	9.08%	9.03%
25–34	15.34%	24.42%	24.40%	19.38%	23.75%	23.75%
35–44	20.28%	20.88%	20.73%	19.38%	19.96%	19.94%
45–54	20.69%	17.95%	17.93%	19.38%	17.11%	17.10%
55–75	22.81%	27.56%	27.54%	20.94%	30.10%	30.19%
Gender (male, %)	55.00%	51.36%	51.35%	48.98%	52.35%	52.19%
Marital status (married, %)	73.56%	78.53%		72.06%	79.41%	
*Socioeconomic status*						
Education attainment (postsecondary and above, %)	67.61%	67.95%		54.82%	51.65%	
Years of schooling (2–23, mean)	15.05 (0.09)	15.11 (0.10)		14.27 (0.10)	14.07 (0.11)	
Employment (currently working, %)	65.79%	67.50%		74.20%	67.61%	
Occupation (professional/managerial, %)	33.20%	34.11%		21.52%	23.39%	
Work experience (years, mean)	14.32 (0.39)	15.25 (0.40)		11.27 (0.32)	12.65 (0.35)	
Personal income (above RMB 90,000 p.a., %)	50.76%	52.22%		56.57%	58.65%	
Household wealth index (0–7, mean)	4.23 (0.04)	4.21 (0.04)		4.44 (0.03)	4.44 (0.35)	
*K6 psychological distress* (0–12, mean) ^c^	4.34 (0.114)	4.23 (0.12)		4.22 (0.10)	4.19 (0.11)	
*Hukou*						
Local hukou (Beijing/Shanghai/Guangzhou/Shenzhen) (%)	67.21%	68.11%		92.02%	92.15%	
Urban hukou (%)	51.26%	52.39%		43.14%	42.85%	
*Health insurance* (%)						
Urban employee basic medical insurance or government insurance	72.45%	75.18%		65.14%	66.96%	
Others	27.55%	24.82%		34.86%	33.04%	

Standard errors in parentheses.

^a^ No. of respondents (Beijing) = 991. ^b^ No. of respondents (Shanghai) = 1027. ^c^ No. of respondents (Beijing) = 983. No. of respondents (Shanghai) = 1026. Eight respondents from Beijing and one respondent from Shanghai did not answer any of the six items in the K6 psychological distress scale.

**Table 4:** Descriptive statistics of the study sample (Guangzhou and Shenzhen)

Variables	Guangzhou ^a^			Shenzhen ^b^		
	Mean/percentage	Poststratification weighted mean/percentage	2020 China Census	Mean/percentage	Poststratification weighted mean/percentage	2020 China Census
*Demographic characteristics*						
Age (18–75, mean)	40.67 (0.48)	39.84 (0.45)		38.63 (0.43)	36.77 (0.36)	
Age groups (%)						
18–24	20.00%	16.13%	16.12%	18.42%	13.79%	13.97%
25–34	20.78%	28.09%	27.96%	24.77%	35.61%	35.59%
35–44	20.00%	20.53%	20.64%	19.86%	23.64%	23.63%
45–54	19.61%	17.81%	17.91%	19.75%	16.83%	16.47%
55–75	19.61%	17.45%	17.37%	17.20%	10.13%	10.34%
Gender (male, %)	50.49%	52.77%	52.82%	44.42%	54.71%	55.25%
Marital status (married, %)	75.10%	76.03%		71.85%	71.06%	
*Socioeconomic status*						
Education attainment (postsecondary and above, %)	60.29%	62.76%		70.73%	73.95%	
Years of schooling (2–23, mean)	14.49 (0.10)	14.65 (0.10)		15.30 (0.08)	15.47 (0.08)	
Employment (currently working, %)	78.43%	80.88%		86.49%	88.43%	
Occupation (professional/managerial, %)	16.37%	17.00%		25.38%	27.66%	
Work experience (years, mean)	9.58 (0.25)	9.49 (0.24)		9.12 (0.27)	9.27 (0.25)	
Personal income (above RMB 90,000 p.a., %)	58.63%	59.56%		63.66%	66.25%	
Household wealth index (0–7, mean)	4.30 (0.03)	4.31 (0.03)		4.01 (0.04)	4.10 (0.04)	
*K6 psychological distress* (0–12, mean) ^c^	5.19 (0.114)	5.20 (0.12)		3.81 (0.106)	4.03 (0.12)	
*Hukou*						
Local hukou (Beijing/Shanghai/Guangzhou/Shenzhen) (%)	92.65%	93.08%		74.00%	72.83%	
Urban hukou (%)	43.04%	43.52%		42.17%	41.57%	
*Health insurance type* (%)						
Urban employee basic medical insurance or government insurance	61.28%	62.05%		75.33%	79.87%	
Others	38.73%	37.95%		24.67%	20.13%	

Standard errors in parentheses.

^a^ No. of respondents (Guangzhou) = 1020. ^b^ No. of respondents (Shenzhen) = 977. ^c^ No. of respondents (Guangzhou) = 1020. No. of respondents (Shenzhen) = 976. One respondent from Shenzhen did not answer any of the six items in the K6 psychological distress scale.

### Data Analysis

Mixed logit regressions were used to capture preference heterogeneity across the sample (van den Broek-Altenburg & Atherly, 2020). These models include random parameters (or coefficients) that vary across individuals, allowing for identification of individual-level preference differences. By permitting random variation in parameters, the model accounts for diverse preferences and reveals systematic patterns of variation (Bridges et al., 2011). The out-of-pocket cost, a monetary factor, was treated as nonrandom to facilitate the derivation of the willingness-to-pay distribution (Bliemer & Rose, 2013), assuming homogeneous preferences for service costs. All other attributes were modeled as random variables, following a normal distribution. Dummy coding was applied to all attribute levels, except for cost, which was treated as continuous.

Using the mixed logit regression model, we calculated the relative importance of each attribute to assess its impact on the choice decision. Relative importance indicates the weight of each attribute in the overall utility, calculated by dividing the range of utility values for each attribute by the total sum of ranges across all attributes (Lancsar et al., 2007). Both the mixed logit regressions and relative importance estimations were performed in R.

While mixed logit regressions capture preference heterogeneity, they do not identify its underlying sources. To address this, we applied latent class modeling to further explore heterogeneous effects on choices. Latent class modeling identifies distinct classes within a sample, assuming individuals within the same class share identical preference weights that differ from those in other classes (Hauber et al., 2016; McGrady et al., 2021). We estimated the latent class preference weights using the conditional logit model in STATA, incorporating respondents’ choices and socioeconomic characteristics to determine the likelihood of membership in each class.

### Ethics

The study received ethical approval from The [redacted] University Institutional Review Board before data collection commenced. Participants provided written informed consent, and their anonymity was safeguarded throughout the study. All data handling, management, and storage adhered to institutional protocols for research involving human subjects.

## Results

### Mixed Logit Regressions and Relative Importance

The mixed logit regressions reveal preference heterogeneity in mental health service utilization across the study sample. Tables 5 and 6 present the regression results for hypothetical scenarios involving potential patients and family members, respectively. The significance of the parameters indicates the influence of different attributes on service choices. Specifically, the coefficient for the cost variable represents the change in utility associated with an additional RMB 100 expenditure, while the coefficients for other attributes reflect the change in utility relative to each attribute’s reference level.

**Table 5:** Mixed logit regression model (patients)

Attributes	Levels	Mean coefficients			
		Beijing	Shenzhen	Shanghai	Guangzhou
Service providers	Community health service center/station	0.674*** (0.055)	0.288*** (0.052)	0.169*** (0.049)	0.179*** (0.050)
	Social service organization	0.510*** (0.052)	0.315*** (0.048)	0.123** (0.046)	0.118* (0.048)
	Public hospital	0.722*** (0.064)	0.283*** (0.059)	0.152** (0.056)	0.194*** (0.058)
	Private clinic/hospital (reference)	–	–	–	–
Recommendation sources	Public education/promotion programs	0.169*** (0.042)	0.051 (0.038)	0.009 (0.037)	−0.003 (0.038)
	Friends/family	0.169*** (0.044)	0.068 (0.040)	−0.085* (0.039)	−0.005 (0.041)
	Past users	0.285*** (0.047)	0.138** (0.044)	0.043 (0.042)	0.067 (0.043)
	Health professionals (reference)	–	–	–	–
Service formats	Online/mobile app	0.002 (0.033)	−0.026 (0.029)	−0.093** (0.029)	−0.116*** (0.030)
	Telephone/hotline	0.106** (0.033)	0.032 (0.030)	−0.011 (0.029)	0.003 (0.030)
	Face-to-face/in-person (reference)	–	–	–	–
Individual/group consultations/treatments	Individual consultation/treatment	0.173*** (0.033)	0.131*** (0.030)	0.010 (0.029)	0.032 (0.030)
	Group consultation/treatment	0.162*** (0.045)	0.072 (0.042)	−0.015 (0.040)	−0.021 (0.041)
	Choice of individual/group consultation/treatment (reference)	–	–	–	–
Intervention types	Alternative treatments	0.234*** (0.049)	0.108* (0.045)	0.132** (0.043)	0.159*** (0.045)
	Lecture/course	0.058 (0.042)	0.080* (0.039)	0.037 (0.037)	0.095* (0.039)
	Psychological counseling/therapies	0.218*** (0.049)	0.074 (0.046)	0.139** (0.044)	0.166*** (0.046)
	Medication (reference)	–	–	–	–
Out-of-pocket costs per visit (in RMB 100 s)		−0.268*** (0.014)	−0.148*** (0.013)	−0.071*** (0.012)	−0.059*** (0.013)
Family involvement	Not always involved	0.227*** (0.025)	0.096*** (0.022)	0.047* (0.022)	0.025 (0.022)
	Always involved (reference)	–	–	–	–
Opting out		−4.538*** (0.212)	−6.898*** (0.331)	−4.089*** (0.165)	−3.878*** (0.169)
Model fit	Akaike Information Criterion	18401.79	17901.81	20545.23	20296.58
	Log Likelihood	−9,169.89	−8,919.90	−10,241.62	−10,117.29
No. of respondents		991	977	1027	1020
Total observations		11,892	11,724	12,324	12,240

Standard errors in parentheses. * *p* < 0.05; ** *p* < 0.01; *** *p* < 0.001.

**Table 6:** Mixed logit regression model (family members)

Attributes	Levels	Mean coefficients			
		Beijing	Shenzhen	Shanghai	Guangzhou
Service providers	Community health service center/station	0.364*** (0.054)	0.171*** (0.048)	0.144** (0.045)	0.071 (0.048)
	Social service organization	0.330*** (0.064)	0.153** (0.059)	0.144** (0.054)	0.063 (0.058)
	Public hospital	0.344*** (0.070)	0.172** (0.065)	0.124* (0.060)	0.033 (0.064)
	Private clinic/hospital (reference)	–	–	–	–
Recommendation sources	Public education/promotion programs	−0.036 (0.048)	−0.046(0.044)	0.027 (0.041)	−0.013 (0.043)
	Friends/family	0.025 (0.054)	0.012 (0.052)	0.104* (0.048)	0.039 (0.050)
	Past users	0.019 (0.049)	−0.036 (0.046)	0.099* (0.043)	0.036 (0.045)
	Health professionals (reference)	–	–	–	–
Service formats	Online/mobile app	0.114** (0.040)	0.010(0.037)	−0.009 (0.035)	−0.002 (0.036)
	Telephone/hotline	0.045 (0.052)	−0.041 (0.049)	−0.049 (0.045)	0.048 (0.048)
	Face-to-face/in-person (reference)	–	–	–	–
Individual/group consultations/treatments	Individual consultation/treatment	0.152*** (0.040)	−0.025(0.036)	−0.006 (0.034)	0.068 (0.036)
	Group consultation/treatment	0.082(0.050)	0.010 (0.048)	−0.036 (0.044)	−0.003(0.046)
	Choice of individual/group consultation/treatment (reference)	–	–	–	–
Intervention types	Alternative treatments	0.011 (0.049)	−0.036 (0.044)	−0.047 (0.042)	−0.054 (0.044)
	Lecture/course	0.055 (0.060)	−0.020 (0.061)	0.021 (0.054)	−0.003 (0.057)
	Psychological counseling/therapy	0.171** (0.058)	0.031 (0.058)	0.102* (0.051)	0.036 (0.055)
	Medication (reference)	–	–	–	–
Out-of-pocket costs per visit (in RMB 100 s)		−0.211*** (0.012)	−0.120*** (0.011)	−0.066*** (0.010)	−0.049*** (0.011)
Family involvement	Not always involved	0.009 (0.027)	−0.029 (0.024)	−0.068** (0.023)	−0.118*** (0.024)
	Always involved (reference)	–	–	–	–
Opting out		−5.309*** (0.243)	−6.875*** (0.332)	−4.308*** (0.181)	−4.433*** (0.189)
Model fit	Akaike Information Criterion	17982.3	17795.05	20226.89	19926.59
	Log Likelihood	−8,960.15	−8,866.53	−10,082.45	−9,932.30
No. of respondents		991	977	1027	1020
Total observations		11,892	11,724	12,324	12,240

Standard errors in parentheses. * *p* < 0.05; ** *p* < 0.01; *** *p* < 0.001.

#### Potential Patients

Across all four cities, potential patient respondents placed higher value on community health service agencies, social service organizations, and public hospitals compared to other providers. In Shenzhen, respondents were more likely to select services recommended by previous users rather than those referred by health professionals, whereas in Beijing, professional recommendations were the least favored option. In Shanghai and Guangzhou, in-person services were preferred over online or mobile-based alternatives. Alternative treatments and psychological counseling had a significant positive impact on utility for respondents in Beijing, Shanghai, and Guangzhou. In both Beijing and Shenzhen, lower levels of family involvement increased the likelihood of seeking professional help. Additionally, the strongly negative coefficient for cost indicates that higher expenses significantly deter potential patients from utilizing professional services.

**Fig. 2:**
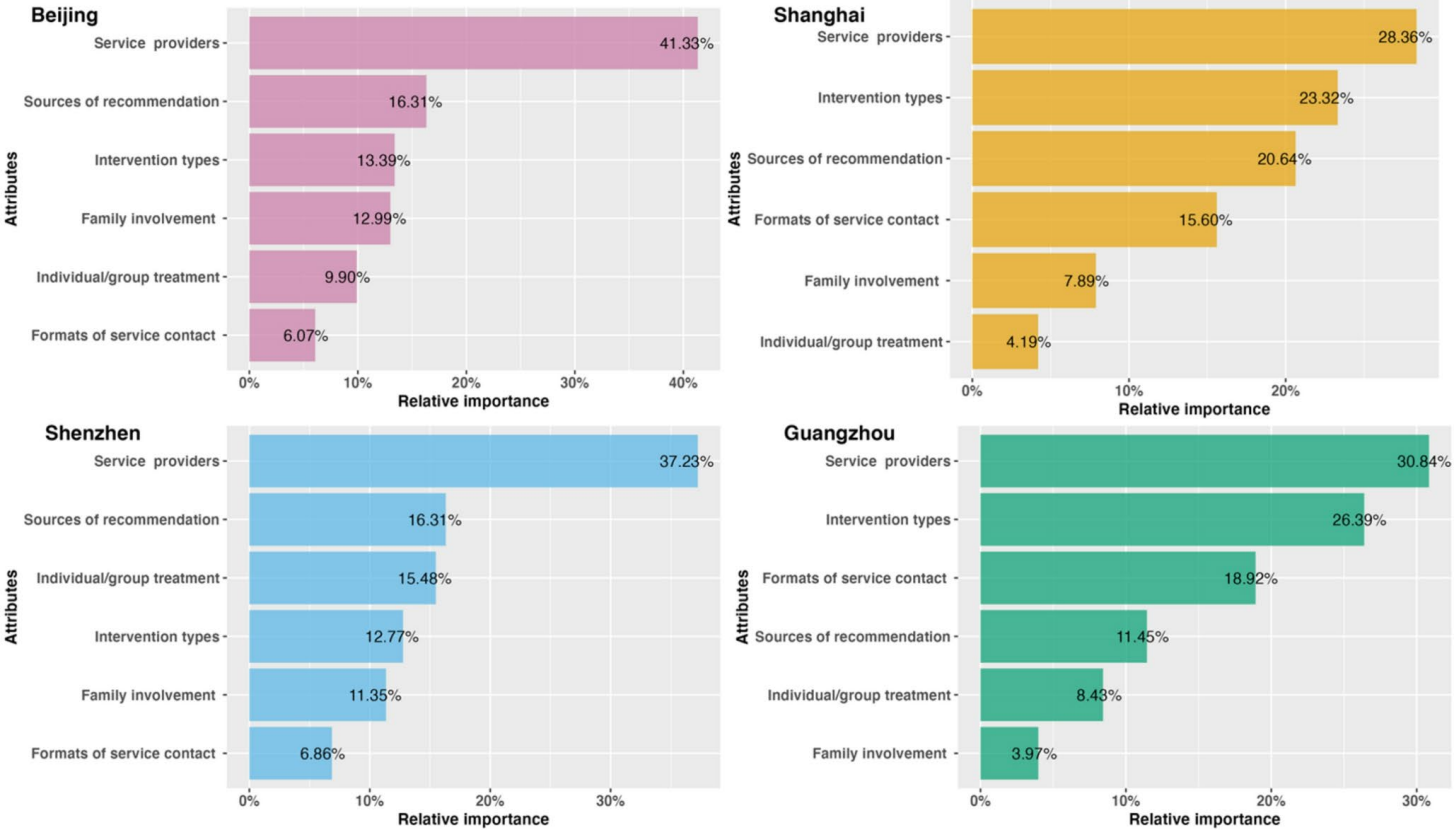
Relative importance (patients)

Figure 2 illustrates the relative importance of attributes for potential patients. Across all four cities, service providers had the greatest influence on respondents’ choices. In Shanghai and Guangzhou, the type of intervention ranked as the second most impactful factor, while family involvement and individual/group treatment had the least influence. In contrast, respondents in Beijing and Shenzhen prioritized the source of recommendation as the second most important attribute, with the format of service contact being the least significant.

#### Family Members

Most family member respondents, except those from Guangzhou, preferred community health facilities, social service organizations, and public hospitals over private facilities for relatives experiencing mild psychiatric symptoms. Respondents in Beijing showed a preference for counseling and therapy services delivered individually via Internet-based platforms. In contrast, those in Guangzhou and Shanghai favored services that involved family participation throughout the treatment process. As expected, respondents generally believed that lower-cost services, assuming all other factors remained constant, were more likely to be utilized by their family members.

**Fig. 3:**
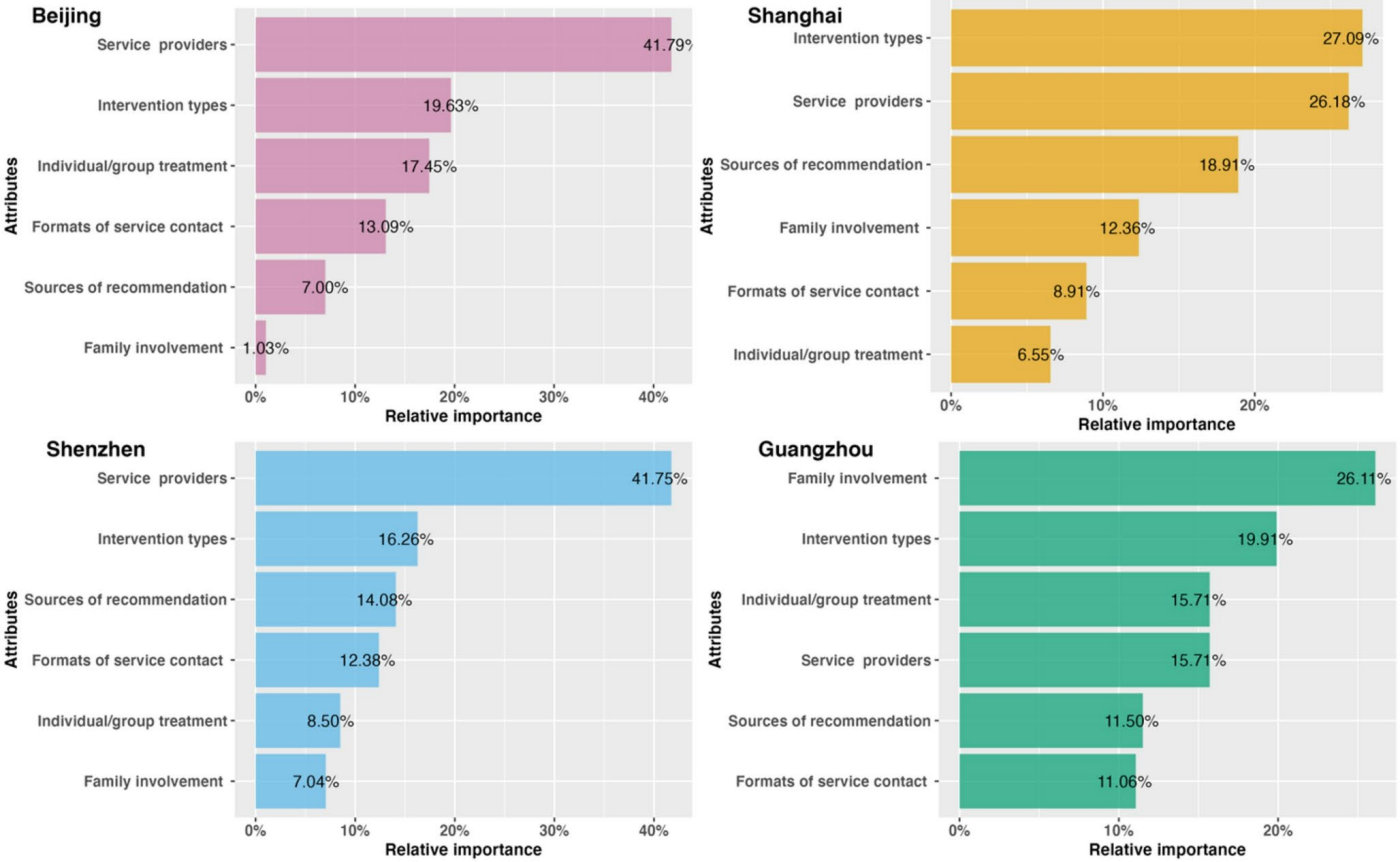
Relative importance (family members)

Figure 3 shows the relative importance of each attribute in the family-member scenario. Among respondents from Beijing, Shenzhen, and Shanghai, service providers and intervention types were the most influential factors in decision-making. In contrast, respondents from Guangzhou placed the highest priority on family involvement.

### Latent Class Models

The latent class modeling findings provide insights into how respondents’ preferences for mental health services vary based on their demographic characteristics. Class membership was determined by participants’ preferences for service attributes alongside their socioeconomic factors. Incorporating variables such as age, gender, marital status, education, employment, income, K6 level, insurance type, hukou, and city of residence allowed for a deeper understanding of preference heterogeneity (see Supplement Tables S1-3 and S2-3). While direct comparison of coefficient magnitudes across classes is generally not feasible, utility differences can be interpreted relative to a common reference level (Hauber et al., 2016). The optimal number of classes was selected based on the Bayesian Information Criterion and Akaike Information Criterion from the converged models.

#### Potential Patients

**Fig. 4:**
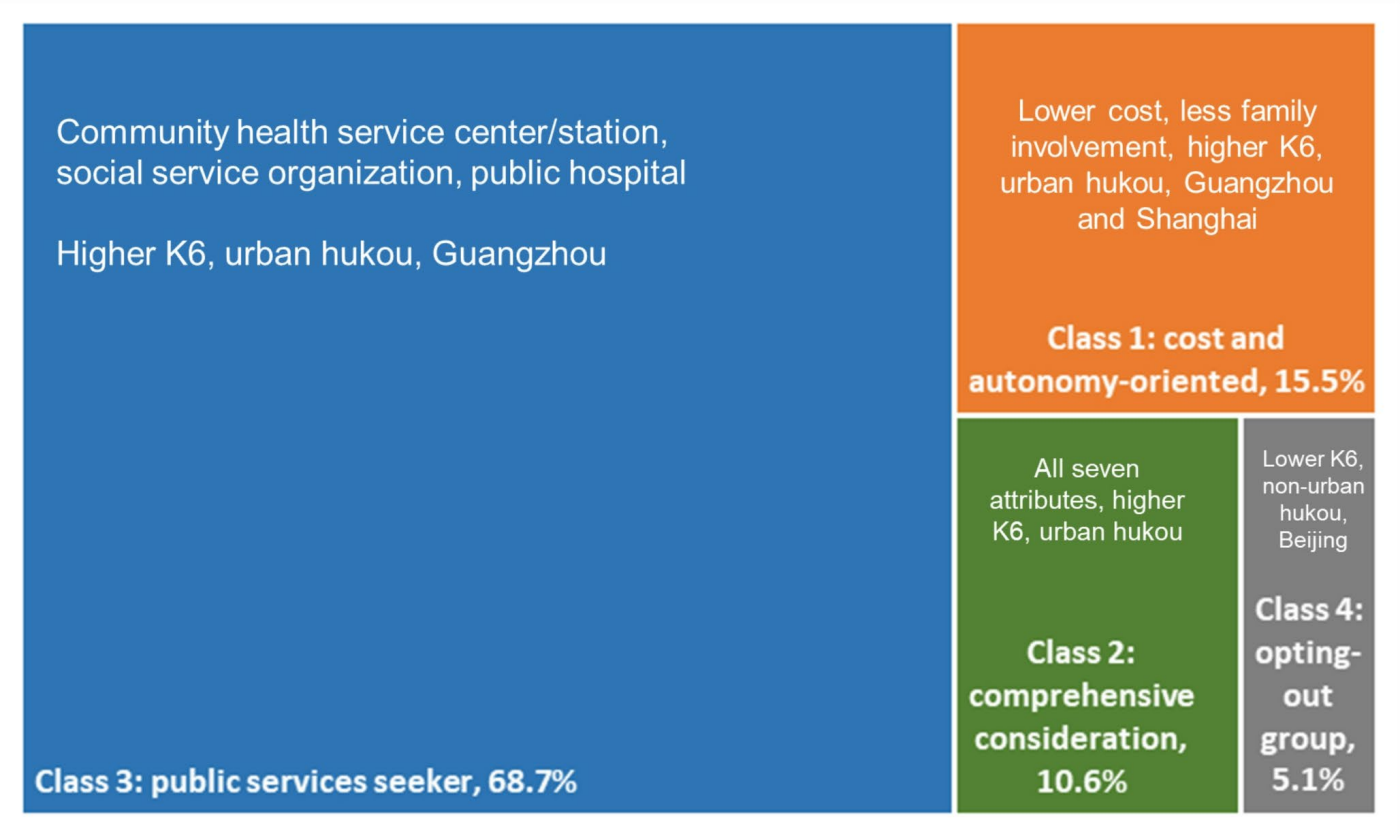
Latent class model (patients)

In the patient scenario, latent class models ranging from two to four classes were estimated, with the optimal number determined using Bayesian Information Criterion, Akaike Information Criterion, and log-likelihood statistics. The four-class model is reported, as it achieved the lowest Bayesian Information Criterion and Akaike Information Criterion values. Figure 4 presents the results for potential patients. Model fit details for the converged latent classes are provided in Supplement Table S1−1. Supplement Table S1−2 reports the latent class regression coefficients for attribute levels within each group, while Supplement Table S1−3 examines the associations between class membership and socioeconomic characteristics. Each class is labeled and described in detail below.

Latent class 1—cost and autonomy-oriented (15.5%): This group prioritizes out-of-pocket costs and minimal family involvement (*p* < 0.001), favoring lower-cost services with greater independence. Compared to the reference Class 4, members report higher psychological distress, are more likely to hold urban hukou, and predominantly reside in Guangzhou and Shanghai.

Latent class 2—comprehensive consideration (10.6%): This group values all seven service attributes, indicating a preference for enhanced mental health services. Members favor public services recommended by health professionals, alternative treatments (individual or group), reduced family involvement, lower costs, and online services. Compared to the reference Class 4, they are more likely to be employed, have higher K6 scores, and hold urban hukou but are less likely to reside in Beijing.

Latent class 3—public services seeker (68.7%): The largest group in the sample, members show a strong preference for initial care from community health facilities, social service organizations, and public hospitals. They also prefer face-to-face treatment services. Compared to other classes, individuals in this group are more likely to experience elevated psychological distress, hold urban hukou, and reside in Guangzhou.

Latent class 4—opting-out group (5.1%): This class is characterized by a strong tendency to opt out of professional services. Members are more likely to reside in Beijing, hold non-urban hukou, and report lower levels of psychological distress.

#### Family Members

**Fig. 5:**
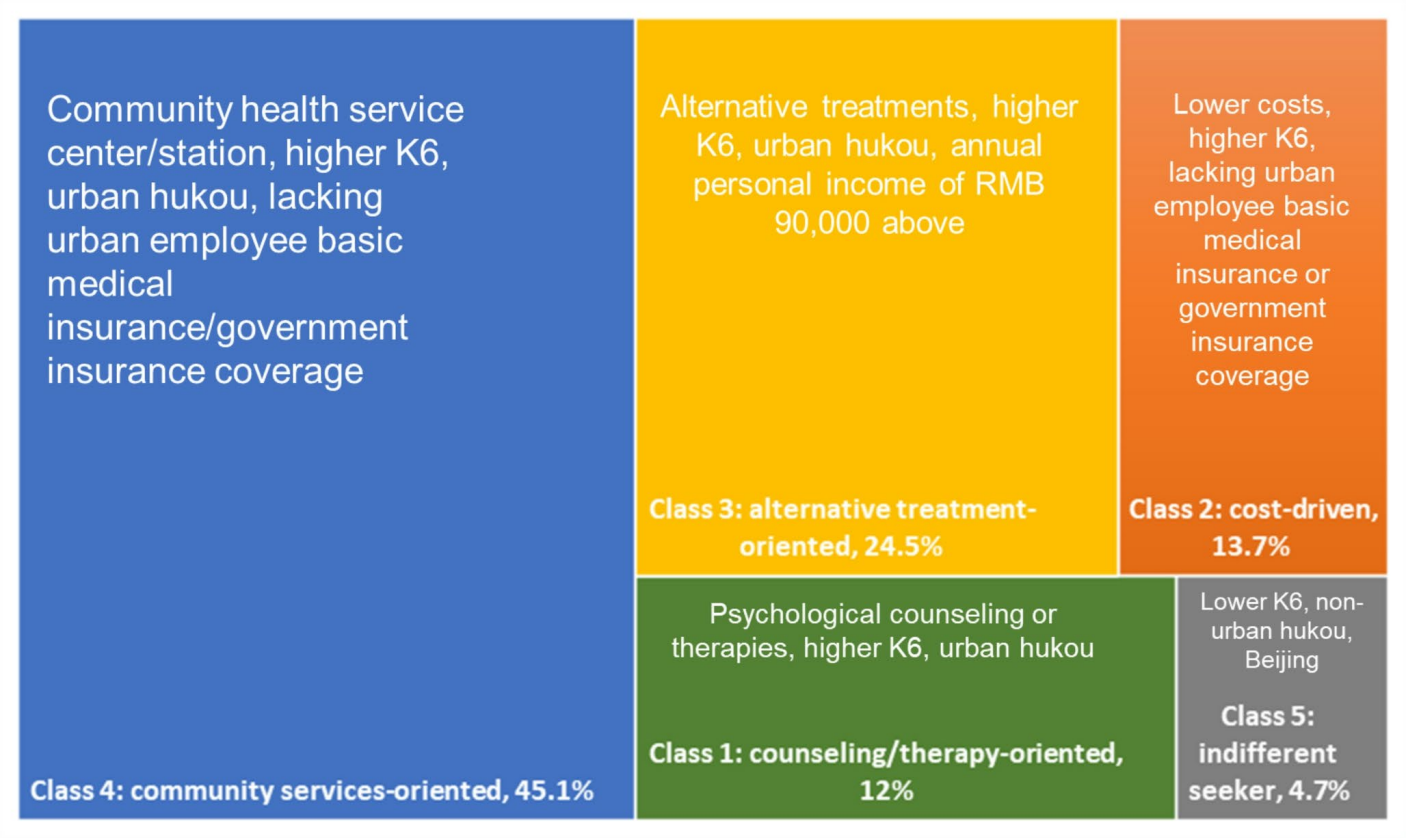
Latent class model (family members)

The converged latent class models for family members suggest between two and five classes. The five-class model, which yields the lowest Bayesian Information Criterion and Akaike Information Criterion values, is considered optimal (Fig. 5). Detailed model fit information for the converged latent classes is provided in Supplement Table S2-1, with the five-class modeling estimates reported in Supplement Tables S2-2 and S2-3.

Latent class 1—counseling/therapy-oriented (12%): This class is characterized by respondents who prefer services offering psychological counseling or therapy for their family members. This group demonstrates a significantly strong preference for counseling and therapy over medication (*p* < 0.001). Compared to Class 5, members are more likely to have higher K6 scores and hold urban hukou but are less likely to possess local hukou.

Latent class 2—cost-driven (13.7%): This class is primarily influenced by cost, which is the only attribute significantly affecting their decision-making (*p* < 0.001). Members of this class prefer services that minimize expenses. They are less likely to have urban employee basic medical insurance or government coverage and are more likely to exhibit higher K6 scores.

Latent class 3—alternative treatment-oriented (24.5%): This class strongly prefers health services offering alternative treatments for their family members (*p* < 0.001). Members show no bias against private clinics or hospitals and slightly favor private over public facilities, such as community health services (*p* < 0.05). They believe services should often exclude family involvement, with cost being a less significant factor in their decision-making. Compared to Class 5, individuals in this class are more likely to have an annual income above RMB 90,000, hold urban hukou, and experience higher levels of psychological distress.

Latent class 4—community services-oriented (45.1%): Representing the largest group, this class prefers family members to seek initial care from community health facilities. Members prioritize health services offering medication or psychological counseling and therapy, with a strong emphasis on ongoing family involvement. Compared to Class 5, individuals in Class 4 tend to have higher K6 scores, are more likely to hold urban hukou, and are less likely to be covered by employee medical insurance or other government insurance programs.

Latent class 5—indifferent seeker (4.7%): This class is distinct in that the coefficient for opting out is not significantly negative (β = 0.173, *p* > 0.05), suggesting that respondents view their family members as indifferent to professional services. Members are more likely to be from Beijing, possess non-urban hukou, and exhibit low levels of psychological distress.

## Discussion

In 2016, the Chinese government launched the Healthy China 2030 initiative, a nationwide strategy aimed at improving population health (Li et al., 2023). Its action plan for the period from 2019 to 2030 emphasizes health promotion, with a particular focus on mental health. The initiative encourages individuals and families to raise awareness of mental health issues and adopt effective stress management practices (Qin et al., 2024; Wang & Liu, 2023).

The government’s efforts extend beyond individual initiatives, encompassing public education campaigns designed to raise awareness of mental health issues, reduce stigma, and promote early intervention (Liang et al., 2018). Simultaneously, there are ongoing efforts to build robust community mental health service networks to provide accessible, localized support (Yu et al., 2018). These initiatives aim to integrate mental health into the broader public health framework (Liang et al., 2018), fostering a society that recognizes mental health as a crucial component of overall well-being.

This study provides valuable insights into the mental health preferences of Chinese adults. A key finding is that the type of service provider is the most significant factor influencing respondents’ decisions to seek professional support for mild psychiatric symptoms. Across all four megacities, participants consistently preferred affordable public services.

This preference for public services is influenced by both socioeconomic factors and the structural characteristics of China’s healthcare system (Qin & Hsieh, 2023; Zhong & Wang, 2021). Public facilities are often perceived as more affordable and reliable, making them accessible to urban residents with limited financial resources (Zhong & Wang, 2021). This preference is further reinforced by significant income disparities, with affordability serving as a key determinant of healthcare utilization (Qin & Hsieh, 2023). Additionally, the long-standing dominance of public institutions has built public trust, making these services the default choice for many individuals (Zhong & Wang, 2021).

The mixed logit regression results reveal notable differences in the preferences of potential patients and their family members, particularly regarding psychological counseling and therapies. Potential patients demonstrated a stronger preference for alternative treatments over medication, whereas family members did not share this inclination.

Culturally, potential patients’ preference for alternative treatments reflects the enduring influence of Traditional Chinese Medicine, which emphasizes natural and holistic approaches (Lu et al., 2022). This preference may also stem from concerns about the side effects and dependency risks associated with psychiatric medications. In contrast, family members tend to favor treatments they perceive as scientifically validated, reflecting the growing acceptance of biomedicine in contemporary Chinese society. These differing perspectives underscore the importance of considering sociocultural dynamics when designing mental health interventions (Chen, 2018; Chen et al., 2024).

The latent class models reveal significant heterogeneity in preferences across the sample, with self-reported K6 scores and hukou type playing key roles in shaping healthcare choices. Specifically, individuals with higher distress levels and urban hukou were more likely to prefer public services over private ones.

The hukou system, a sociopolitical framework governing household registration, significantly influences healthcare access and preferences in China (Chen, 2018). Urban hukou holders typically enjoy greater healthcare benefits and more consistent access to public services compared to their rural counterparts (Li & Chen, 2024). This disparity not only reinforces the preference for public facilities among urban residents but also underscores ongoing inequities within the healthcare system (Zhang et al., 2022). Addressing these inequities is essential for ensuring equitable access to mental health services across all populations.

Beyond the megacities studied, the findings have broader implications for the design of mental health services both within China and internationally. In rural and smaller urban areas, where healthcare resources are limited and stigma remains a significant barrier (Yu et al., 2018; Zhang et al., 2022), service preferences may differ considerably. Tailoring strategies to these contexts requires addressing local sociocultural, economic, and systemic factors. Globally, the findings highlight the critical roles of affordability and public trust in expanding mental health access, particularly in low- and middle-income countries facing similar healthcare disparities. By aligning interventions with the specific needs of underserved regions, policymakers can create more equitable mental healthcare systems, bridging gaps in both accessibility and quality.

These findings provide critical insights for optimizing resource allocation within China’s mental healthcare system. Policymakers should prioritize strategies that enhance the cost-effectiveness of public mental health services while developing interventions tailored to the needs of diverse socioeconomic groups (Dev et al., 2020). Key recommendations include increasing investment in community health facilities, especially in underserved regions, to address access and resource disparities. Additionally, comprehensive stigma-reduction campaigns are crucial for encouraging help-seeking behaviors and overcoming sociocultural barriers. Expanding digital mental health platforms, such as telemedicine and AI-based counseling services (Bazarkina et al., 2023), offers a promising solution to bridge service gaps, particularly in remote or resource-limited areas. Together, these measures can improve accessibility, equity, and the overall quality of mental health services for diverse populations.

Our study highlights the value of the discrete choice experiment methodology in shaping patient-centered policy. Discrete choice experiments effectively identify the key attributes of health services that patients prioritize, offering actionable insights for designing targeted interventions (McGrady et al., 2021). Their adaptability ensures they remain applicable across various healthcare settings, allowing interventions to stay responsive to evolving patient needs. By incorporating the findings from discrete choice experiments, policymakers can allocate resources more efficiently and direct investments toward services and treatments that align with patient preferences (Lancsar & Louviere, 2008).

Integrating patient preferences into healthcare design enhances patient engagement and adherence to treatment plans (Chen et al., 2024). When patients feel their preferences are valued, they are more likely to actively participate in their care (Seghers et al., 2022). Furthermore, discrete choice experiments identify key areas for quality improvement by revealing aspects of care that patients prioritize. This enables the development of targeted initiatives that align with patient expectations and needs (Bickman et al., 2016).

Expanding the application of discrete choice experiments to rural areas and smaller cities in China could reveal unique local barriers and service preferences. This approach would aid in developing equitable mental healthcare systems by addressing regional disparities and aligning interventions with the diverse needs of populations across the country (Zhang et al., 2022). Such efforts are crucial for building a more inclusive and accessible mental health framework that bridges gaps in care and promotes nationwide health equity.

### Reflexivity

While this study is based on a large sample from Beijing, Shanghai, Guangzhou, and Shenzhen, its findings may not be fully generalizable to other regions, particularly rural areas or smaller cities with less developed mental health services and infrastructure. The emphasis on megacities, which have more advanced mental health services, could lead to an overestimation of service availability and utilization preferences compared to the national average. Additionally, the reliance on self-reported data on psychological distress and preferences introduces the risk of recall and social desirability biases, as respondents may underreport or overreport their distress levels or preferences due to stigma or challenges in accurately assessing their experiences. Furthermore, the cross-sectional design captures preferences and attitudes at a single point in time, without accounting for potential changes over time driven by evolving mental health policies, increasing public awareness, or personal experiences with mental health services.

Another limitation of this study is the potential bias introduced by Internet-based surveys. Respondents recruited through online platforms may not fully represent the broader population, leading to the overrepresentation or underrepresentation of certain groups. Although strategies like weighting adjustments, targeted recruitment, and mixed survey modes were employed to mitigate these risks, individuals with limited Internet access or digital literacy may have been excluded. Furthermore, this study focused exclusively on preferences for first-contact mental health services, which limits its ability to capture residents’ broader care-seeking behaviors throughout the entire healthcare pathway. While mixed logit regressions and latent class models are effective in categorizing respondents based on influencing factors, these quantitative methods are less suited to uncovering the deeper emotional and cognitive reasons behind decision-making. Previous studies have shown that preferences can be shaped by attitudes toward risk and uncertainty (Bridges et al., 2011). In the context of mental health help-seeking, cultural beliefs and stigma play a significant role in shaping individuals’ willingness to seek help and their preferences for services.

Future research should focus on improving sample representativeness to better capture the diverse needs and preferences of populations across various regions. One effective approach is integrating mixed-mode data collection methods, which combine online surveys with offline recruitment in areas with limited Internet access or low digital literacy. This can help ensure the inclusion of underserved groups, such as rural residents or individuals with lower socioeconomic status, who are often underrepresented in online studies. Additionally, expanding research to smaller cities and rural areas is crucial to identify differences in mental health service needs and preferences compared to megacities. These regions may face unique barriers, such as geographic isolation, limited resources, and distinct cultural perceptions of mental health. Investigating these factors will provide valuable insights for developing tailored policies and interventions. Longitudinal research in these contexts would also deepen our understanding of how mental health needs and preferences evolve, especially in response to changes in social and healthcare systems.

## Conclusion

In Mainland China, individuals with mental disorders face numerous challenges, including limited access to medical treatments, long-term medications, and rehospitalization. Additional concerns include fears of relapse, inadequate rehabilitation programs, and financial hardship. Despite nationwide efforts to promote mental health services, significant regional disparities persist. Even in megacities like Beijing, healthcare resources are concentrated in specific districts, resulting in substantial variation in service availability and quality (Chen, 2018). Across the country, especially in rural areas, the demand for mental healthcare far exceeds the supply, while treatment rates for mental illnesses have declined in recent years (Zhang et al., 2022). Addressing these disparities and improving the accessibility and quality of mental health services are crucial to enhancing the well-being of individuals with mental illnesses. Precision mental health practices, which tailor services to the specific preferences and needs of service users, offer a promising solution. By aligning interventions with individual preferences, precision mental health can ensure more effective and sustainable outcomes, ultimately bridging existing gaps and transforming China’s mental health landscape.

## Electronic Supplementary Material

Below is the link to the electronic supplementary material.

Supplementary Material 1

## Data Availability

Data will be made available upon request.
