# Supplementary material for: Precision Mental Healthcare: Identifying Service Preferences Through Discrete-Choice Experiments in Chinese Megacities
Source: Adm Policy Ment Health. 2025 Apr 22;52(4):619–39. doi: 10.1007/s10488-025-01444-z (PMC12310768; doi:10.1007/s10488-025-01444-z)
Supplement: Supplementary file 1 — Supplementary Material 1 [file 10488_2025_1444_MOESM1_ESM.docx]

**Supplementary material for the paper “Precision mental healthcare: Identifying service preferences through discrete-choice experiments in Chinese megacities”**

**Robustness checks: Propensity score weighting**

While online surveys offer several advantages, they may fail to accurately represent the target population, potentially leading to overrepresentation or underrepresentation of certain groups. To address selection biases, we employed methods from Valliant and Dever (2011) to estimate propensity scores and assign weights to nonprobability samples. Using data from the 2022 Beijing Area Study (BAS) as a probability reference sample, we computed pseudo-weights for our current Beijing sample (N = 991) through propensity modeling, ensuring a more representative analysis. The weight calculation process involved the following steps:

1. Merge samples: Combined the BAS probability sample with the nonprobability sample from Beijing, assigning a weight of 1 to all nonprobability units.
2. Estimate propensity scores: Used weighted logistic regression to calculate propensity scores and generate weights for the nonprobability sample, incorporating covariates such as age, gender, ethnicity, education level, and marital status.
3. Extract nonprobability samples: Isolated nonprobability samples with their corresponding weights.
4. Poststratification adjustment: Adjusted the nonprobability sample to align with the target population’s distribution on key variables, including age and gender.
5. Standardize weights: Standardized the weights to produce the final values for analysis.

Table S3 provides descriptive statistics for the study sample after applying propensity score weighting. The mixed logit regression results, adjusted with propensity score weighting, are presented in Table S4 for patients and Table S5 for family members.

Table S1-1. Model fit information on the converged latent class models (patients)

| Number of classes | Observations | Log(L) | df | AIC | BIC |
| --- | --- | --- | --- | --- | --- |
| 2 | 144,180 | -39275.31 | 50 | 78650.61 | 79144.55 |
| 3 | 144,180 | -38573.46 | 81 | 77308.93 | 78109.11 |
| 4 | 144,180 | -36851.09 | 118 | 73938.18 | 75103.88 |

Table S1-2. Latent class model (patients)

| Attributes | Levels | Class 1: cost and autonomy-oriented | Class 2: comprehensive consideration | Class 3: public services seeker | Class 4: opting-out group |
| --- | --- | --- | --- | --- | --- |
| Service providers | Community health service center/station | 0.096 | 4.239*** | 0.122*** | 0.167 |
|  | Social service organization | 0.036 | 4.673*** | 0.106*** | 0.881*** |
|  | Public hospital | 0.160* | 4.094*** | 0.099** | 0.29 |
|  | Private clinic/hospital (reference) | – | – | – | – |
| Recommendation sources | Public education/promotion programs | 0.059 | -1.153*** | -0.016 | -0.450* |
|  | Friends/family | 0.021 | -4.401*** | -0.062* | 0.142 |
|  | Past users | 0.099 | -0.911*** | 0.004 | 0.371* |
|  | Health professionals (reference) | – | – | – | – |
| Service formats | Online/mobile app | -0.051 | 1.490*** | -0.178*** | 0.393* |
|  | Telephone/hotline | 0.051 | -2.609*** | -0.038* | -0.163 |
|  | Face-to-face/in-person (reference) | – | – | – | – |
| Individual/group consultations/treatments | Individual consultation/treatment | -0.063 | 0.996*** | 0.014 | 0.441*** |
|  | Group consultation/treatment | -0.014 | 1.300*** | -0.107*** | 0.172 |
|  | Choice of individual/group sessions (reference) | – | – | – | – |
| Intervention types | Alternative treatments | 0.117* | 5.646*** | 0.013 | 0.245 |
|  | Lecture/course | -0.027 | 0.214 | 0.005 | 0.027 |
|  | Psychological counseling/therapy | 0.150* | -2.572*** | 0.046 | 0.660** |
|  | Medication (reference) | – | – | – | – |
| Out-of-pocket costs per visit (in RMB 100s) | | -0.112*** | -3.477*** | 0.015 | -4.137*** |
| Family involvement | Not always involved | 0.111*** | 0.460*** | 0.043** | 0.595*** |
|  | Always involved (reference) | – | – | – | – |
| Opting out | | -0.480*** | -11.371*** | -4.485*** | 1.138* |
| Class share | | 15.5% | 10.6% | 68.7% | 5.1% |
| *** p < 0.001, ** p < 0.01, * p < 0.05. | | | | | |

Table S1-3. Class membership in latent class model (patients)

| Class membership/description | Class 1: cost and autonomy-oriented | Class 2: comprehensive consideration | Class 3: public services seeker | Class 4: opting-out group |
| --- | --- | --- | --- | --- |
| Age | -0.004 | 0.003 | 0.003 | Reference |
| Gender (male) | 0.435* | 0.197 | 0.108 |  |
| Marital status (married) | 0.195 | 0.104 | 0.023 |  |
| Education attainment (postsecondary and above) | -0.386 | -0.837* | -0.315 |  |
| Years of schooling | -0.02 | 0.025 | -0.062 |  |
| Employment (currently working) | 0.306 | 0.751** | 0.444* |  |
| Occupation (professional/managerial) | 0.364 | -0.177 | 0.244 |  |
| Work experience (years) | -0.016 | -0.006 | -0.016 |  |
| Personal income (above RMB 90,000 p.a.) | 0.011 | 0.252 | 0.428* |  |
| Household wealth index (0–7, mean) | -0.091 | 0.077 | 0.111 |  |
| K6 psychological distress | 0.198*** | 0.193*** | 0.264*** |  |
| Local hukou (BJ/SH/GZ/SZ) | -0.143 | -0.447* | 0.094 |  |
| Urban hukou | 0.622** | 0.959*** | 1.047*** |  |
| UEBMI /government insurance | -0.451* | -0.222 | -0.37 |  |
| City of residence-Beijing | -0.717** | -1.123*** | -1.351*** |  |
| City of residence-Guangzhou | 2.100*** | 0.437 | 1.104** |  |
| City of residence-Shanghai | 1.305*** | -0.109 | 0.184 |  |
| Constant | 0.954 | -0.204 | 1.937* |  |
| *** p < 0.001, ** p < 0.01, * p < 0.05. | | | | |

Table S2-1. Model fit information on the converged latent class models (family members)

| Number of classes | Observations | Log(L) | df | AIC | BIC |
| --- | --- | --- | --- | --- | --- |
| 2 | 144,180 | -38909.98 | 50 | 77919.95 | 78413.89 |
| 3 | 144,180 | -37051.12 | 84 | 74270.24 | 75100.06 |
| 4 | 144,180 | -36044.03 | 118 | 72324.07 | 73489.77 |
| 5 | 144,180 | -35832.64 | 152 | 71969.28 | 73470.86 |

Table S2-2. Latent class model (family members)

| Attributes | Levels | Class 1: counseling/therapy-oriented | Class 2: cost-driven | Class 3: alternative treatment-oriented | Class 4: community services-oriented | Class 5: indifferent seeker |
| --- | --- | --- | --- | --- | --- | --- |
| Service providers | Community health service center/station | 0.18 | 0.09 | -0.169* | 0.197*** | 0.099 |
|  | Social service organization | 0.322 | 0.084 | -0.081 | 0.098 | -0.079 |
|  | Public hospital | 0.597 | 0.063 | -0.058 | 0.047 | -0.066 |
|  | Private clinic/hospital (reference) | – | – | – | – | – |
| Recommendation sources | Public education/promotion programs | 0.859*** | -0.017 | 0.142* | -0.192*** | 0.379 |
|  | Friends/family | -0.423 | -0.013 | 0.205* | -0.093 | 0.137 |
|  | Past users | 0.504 | -0.045 | 0.156 | -0.016 | 0.057 |
|  | Health professionals (reference) | – | – | – | – | – |
| Service formats | Online/mobile app | 0.17 | -0.084 | -0.160* | 0.039 | -0.082 |
|  | Telephone/hotline | -0.121 | 0.008 | -0.156 | -0.096* | 0.198 |
|  | Face-to-face/in-person (reference) | – | – | – | – | – |
| Individual/group consultations/treatments | Individual consultation/treatment | 0.505* | 0.092 | -0.092 | 0.028 | -0.228 |
|  | Group consultation/treatment | 0.858** | 0.073 | -0.161* | -0.076 | -0.051 |
|  | Choice of individual/group sessions (reference) | – | – | – | – | – |
| Intervention types | Alternative treatments | 0.720* | -0.013 | 0.327*** | -0.337*** | -0.165 |
|  | Lecture/course | 0.804** | -0.003 | 0.256* | -0.238*** | 0.057 |
|  | Psychological counseling/therapy | 2.285*** | -0.062 | 0.201 | -0.065 | 0.11 |
|  | Medication (reference) | – | – | – | – | – |
| Out-of-pocket costs per visit (in RMB 100s) | | -1.984*** | -0.065*** | 0.014 | 0.015 | -6.508*** |
| Family involvement | Not always involved | -0.006 | -0.01 | 0.495*** | -0.376*** | 0.396** |
|  | Always involved (reference) | – | – | – | – | – |
| Opting out | | -5.063*** | -0.529*** | -3.931*** | -5.192*** | 0.173 |
| Class share | | 12% | 13.70% | 24.50% | 45.10% | 4.70% |

*** p < 0.001, ** p < 0.01, * p < 0.05.

Table S2-3. Class membership in latent class model (family members)

| Class membership | Class 1: counseling/therapy-oriented | Class 2: cost-driven | Class 3: alternative treatment-oriented | Class 4: community services-oriented | Class 5: indifferent seeker |
| --- | --- | --- | --- | --- | --- |
| Age | 0.004 | -0.004 | 0.015 | 0.003 | Reference |
| Gender (male) | 0.408* | 0.552** | 0.044 | 0.222 |  |
| Marital status (married) | 0.38 | 0.033 | -0.145 | -0.132 |  |
| Education attainment (postsecondary and above) | -0.425 | -0.258 | 0.014 | -0.29 |  |
| Years of schooling | 0.125 | 0.07 | -0.033 | 0.047 |  |
| Employment (currently working) | 0.586* | 0.144 | 0.543* | 0.23 |  |
| Occupation (professional/managerial) | -0.148 | 0.12 | -0.036 | 0.179 |  |
| Work experience (years) | -0.012 | -0.018 | -0.009 | -0.021 |  |
| Personal income (above RMB 90,000 p.a.) | 0.036 | 0.09 | 0.578** | 0.396* |  |
| Household wealth index (0–7, mean) | 0.165* | 0.012 | 0.078 | 0.188** |  |
| K6 psychological distress | 0.206*** | 0.222*** | 0.259*** | 0.281*** |  |
| Local hukou (BJ/SH/GZ/SZ) | -0.644** | -0.102 | 0.045 | 0.092 |  |
| Urban hukou | 0.856*** | 0.744*** | 1.004*** | 1.138*** |  |
| UEBMI/government insurance | -0.078 | -0.572** | -0.325 | -0.577** |  |
| City of residence-Beijing | -0.836*** | -0.799** | -1.551*** | -1.169*** |  |
| City of residence-Guangzhou | 0.111 | 1.431*** | 0.459 | 0.662* |  |
| City of residence-Shanghai | -0.046 | 1.147*** | 0.087 | 0.204 |  |
| Constant | -1.253 | -0.073 | 0.014 | 0.673 |  |

*** p < 0.001, ** p < 0.01, * p < 0.05.

Table S3. Descriptive statistics of the study sample with propensity score weighting (Beijing)

| Variables | Beijing ^a^ | | | |
| --- | --- | --- | --- | --- |
|  | Mean/percentage | Poststratification weighted mean/percentage | Propensity-weighted mean/percentage | 2020 China Census |
| *Demographic characteristics* |  |  |  |  |
| Age (18–75, mean) | 41.13 (0.48) | 43.09 (0.49) | 43.28 (0.80) |  |
| Age groups (%) |  |  |  |  |
| 18–24 | 20.89% | 9.20% | 9.41% | 9.41% |
| 25–34 | 15.34% | 24.42% | 24.40% | 24.40% |
| 35–44 | 20.28% | 20.88% | 20.73% | 20.73% |
| 45–54 | 20.69% | 17.95% | 17.93% | 17.93% |
| 55–75 | 22.81% | 27.56% | 27.54% | 27.54% |
| Gender (male, %) | 55.00% | 51.36% | 51.35% | 51.35% |
| Marital status (married, %) | 73.56% | 78.53% | 77.16% |  |
| *Socioeconomic status* |  |  |  |  |
| Education attainment (postsecondary and above, %) | 67.61% | 67.95% | 46.97% |  |
| Years of schooling (2–23, mean) | 15.05 (0.09) | 15.11 (0.10) | 13.51 (0.22) |  |
| Employment (currently working, %) | 65.79% | 67.50% | 75.08% |  |
| Occupation (professional/managerial, %) | 33.20% | 34.11% | 28.09% |  |
| Work experience (years, mean) | 14.32 (0.39) | 15.25 (0.40) | 14.92 (0.62) |  |
| Personal income (above RMB 90,000 p.a., %) | 50.76% | 52.22% | 50.89% |  |
| Household wealth index (0–7, mean) | 4.23 (0.04) | 4.21 (0.04) | 4.18 (0.07) |  |
| *K6 psychological distress* (0–12, mean) ^b^ | 4.34 (0.114) | 4.23 (0.12) | 4.24 (0.23) |  |
| *Hukou* |  |  |  |  |
| Local hukou (BJ/SH/GZ/SZ) (%) | 67.21% | 68.11% | 64.60% |  |
| Urban hukou (%) | 51.26% | 52.39% | 50.68% |  |
| *Health insurance type* (%) |  |  |  |  |
| UEBMI/government insurance ^c^ | 72.45% | 75.18% | 73.10% |  |
| Others | 27.55% | 24.82% | 26.90% |  |
| Standard errors in parentheses.  ^a^ No. of respondents (Beijing) = 991. ^b^ No. of respondents (Beijing) = 983. Eight respondents from Beijing did not answer any of the six items in the K6 psychological distress scale. | | | | |
| ^c^ UEBMI: Urban employee basic medical insurance. | | | | |

Table S4. Mixed logit regression with propensity score weighting (Beijing patients)

| Attributes | Levels | Poststratification weighted estimators | Propensity-weighted estimators |
| --- | --- | --- | --- |
| Service providers | Community health service center/station | 0.674*** (0.055) | 0.524*** (0.036) |
|  | Social service organization | 0.510*** (0.052) | 0.439*** (0.034) |
|  | Public hospital | 0.722*** (0.064) | 0.564*** (0.042) |
|  | Private clinic/hospital (reference) | – | – |
| Recommendation sources | Public education/promotion programs | 0.169*** (0.042) | 0.223*** (0.028) |
|  | Friends/family | 0.169*** (0.044) | 0.128*** (0.027) |
|  | Past users | 0.285*** (0.047) | 0.240*** (0.031) |
|  | Health professionals (reference) | – | – |
| Service formats | Online/mobile app | 0.002 (0.033) | -0.021 (0.021) |
|  | Telephone/hotline | 0.106** (0.033) | 0.097*** (0.021) |
|  | Face-to-face/in-person (reference) | – | – |
| Individual/group consultations/treatments | Individual consultation/treatment | 0.173*** (0.033) | 0.121*** (0.022) |
|  | Group consultation/treatment | 0.162*** (0.045) | 0.034 (0.030) |
|  | Choice of individual/group consultation/treatment (reference) | – | – |
| Intervention types | Alternative treatments | 0.234*** (0.049) | 0.178*** (0.030) |
|  | Lecture/course | 0.058 (0.042) | -0.013 (0.027) |
|  | Psychological counseling/ therapy | 0.218*** (0.049) | 0.179*** (0.031) |
|  | Medication (reference) | – | – |
| Out-of-pocket costs per visit (in RMB 100s) | | -0.268*** (0.014) | -0.202*** (0.009) |
| Family involvement | Not always involved | 0.227*** (0.025) | 0.200*** (0.016) |
|  | Always involved (reference) | – | – |
| Opting out |  | -4.538*** (0.212) | -4.852*** (0.134) |
| Model fit | Akaike Information Criterion | 18401.79 | 18306.17 |
|  | Log Likelihood | -9,169.89 | -9,122.09 |
| No. of respondents (Beijing) | | 991 | 991 |
| Total observations | | 11,892 | 11,892 |
| Standard errors in parentheses. * p < 0.05; ** p < 0.01; *** p < 0.001. | | | |

Table S5. Mixed logit regression with propensity score weighting (Beijing family members)

| Attributes | Levels | Poststratification weighted estimators | Propensity-weighted estimators |
| --- | --- | --- | --- |
| Service providers | Community health service center/station | 0.364*** (0.054) | 0.373*** (0.033) |
|  | Social service organization | 0.330*** (0.064) | 0.276*** (0.039) |
|  | Public hospital | 0.344*** (0.070) | 0.336*** (0.044) |
|  | Private clinic/hospital (reference) | – | – |
| Recommendation sources | Public education/promotion programs | -0.036 (0.048) | -0.042 (0.028) |
|  | Friends/family | 0.025 (0.054) | -0.072* (0.034) |
|  | Past users | 0.019 (0.049) | -0.102*** (0.031) |
|  | Health professionals (reference) | – | – |
| Service formats | Online/mobile app | 0.114** (0.040) | 0.143*** (0.024) |
|  | Telephone/hotline | 0.045 (0.052) | 0.059 (0.032) |
|  | Face-to-face/in-person (reference) | – | – |
| Individual/group consultations/treatments | Individual consultation/treatment | 0.152*** (0.040) | 0.131*** (0.023) |
|  | Group consultation/treatment | 0.082 (0.050) | 0.117*** (0.030) |
|  | Choice of individual/group consultation/treatment (reference) | – | – |
| Intervention types | Alternative treatments | 0.011 (0.049) | 0.167*** (0.030) |
|  | Lecture/course | 0.055 (0.060) | 0.033 (0.040) |
|  | Psychological counseling/therapy | 0.171** (0.058) | 0.146*** (0.037) |
|  | Medication (reference) | – | – |
| Out-of-pocket costs per visit (in RMB 100s) | | -0.211*** (0.012) | -0.197*** (0.008) |
| Family involvement | Not always involved | 0.009 (0.027) | 0.067*** (0.016) |
|  | Always involved (reference) | – | – |
| Opting out |  | -5.309*** (0.243) | -5.815*** (0.174) |
| Model fit | Akaike Information Criterion | 17982.3 | 17892.92 |
|  | Log Likelihood | -8,960.15 | -8,915.46 |
| No. of respondents (Beijing) | | 991 | 991 |
| Total observations | | 11,892 | 11,892 |
| Standard errors in parentheses. * p < 0.05; ** p < 0.01; *** p < 0.001. | | | |
